# Loss of Appetite in Adult Patients: Effectiveness and Safety of an Appetite Stimulating Medication in an Open-Label, Investigator-Initiated Study in India

**DOI:** 10.1155/2022/2661912

**Published:** 2022-01-07

**Authors:** S. Nagaraj

**Affiliations:** Infilife Healthcare Pvt. Ltd., Bengaluru, Karnataka, India

## Abstract

Loss of appetite (LOA) may have a negative impact on a patient's well-being owing to loss of nutrition and associated conditions. The current study assessed the effects of an appetite-stimulating medication containing multivitamins, lysine, and zinc in Indian patients with a history of LOA. Using an investigator-initiated, single-center, open-label, single-arm design, we evaluated the effectiveness and safety of the appetite-stimulating medication (15 mL) in 50 male or female patients (18–55 years old) attending the outpatient department, with a confirmed diagnosis of LOA after two weeks of therapy and assessed the change in Council on Nutrition Appetite Questionnaire (CNAQ) score and safety of the medication after two weeks of treatment. CNAQ scores were presented as mean (standard deviation (SD)). The mean age of patients was 42.1 years, with the majority (66%) being males. At weeks 1 and 2, a statistically significant improvement was observed in the mean CNAQ scores of 25.48 (5.10) and 25.48 (4.29), respectively, vs. baseline (22.08 (2.76); *P* ≤ 0.0001 both). Majority of the patients had CNAQ appetite scores of 17–28 at baseline (94%), week 1 (66%), and week 2 (78%) of treatment. For patients with acute and chronic illness, a statistically significant improvement was observed in the mean CNAQ score at week 1 (26.75 (3.69), *P* = 0.0256; 25.24 (5.33), *P* = 0.0004) and at week 2 (26.63 (3.46), *P* = 0.0027; 25.26 (4.43), *P* ≤ 0.0001) from baseline (21.88 (3.31) and 22.12 (2.69), respectively). No serious adverse events were reported during the study. The study findings suggest that appetite-stimulating medication containing multivitamins, lysine, and zinc could be a suitable treatment option for the management of LOA with no significant safety concerns.

## 1. Introduction

Loss of appetite (LOA) or anorexia is a condition of absence of hunger in patients and can occur due to a wide variety of reasons such as age, acute or chronic disease conditions, and associated medications [1]. LOA can cause nutritional deficiency and lead to associated complications that can negatively impact a patient's health, overall well-being, and quality of life (QoL) [2].

The nature of LOA can be brief or temporary or it can be prolonging and long-lasting. Acute LOA is usually temporary and is caused by illness or digestive concerns, resulting in unintentional loss of body weight and poor appetite due to benign viral or bacterial infections. Chronic LOA usually persists for a longer duration due to severe underlying complications and is observed in aging patients and patients with cancer, chronic kidney disease, anxiety disorders (dementia, depression), cardiac disorders, and attention deficit hyperactivity disorder, amongst others [3–7]. LOA-related weight loss has a negative impact on QoL, morbidity, and mortality. Thus, healthcare providers need an accurate tool to evaluate appetite and predict weight loss in patients. The Council on Nutrition Appetite Questionnaire (CNAQ) score is an 8-item questionnaire derived using the Delphi technique that is used for appetite monitoring and predicting weight loss in both young and older patients [8].

LOA is frequently observed in the older population [9], as well as in young patients [10]. Lack of proper nutrition can lead to nutritional deficiencies in a patient (malnutrition or undernutrition). This results in elder patients becoming immunocompromised, susceptible to infections, and losing weight [3], whereas a negative impact on growth and neurodevelopment is observed in children [11]. In India, LOA is observed in a massive 93% of patients with severe illness. The risk is 1.5 times higher in those with a history of any medical ailment [2]. Understanding the underlying condition of a patient is critical to appropriate management of LOA. Appetite stimulation appears to be a beneficial treatment option for management of LOA. Various appetite-stimulating medications are available that can assist patients with LOA to improve appetite and gain weight, thereby, enhancing quality of life. However, treatment is often initiated only once patient suffers substantial weight loss and nutritional deficiency, indicating a gap in the management of patients with LOA [12, 13]. Besides, there is a need to understand the efficacy profile of such medications regarding appetite and nutrition-related outcomes.

In India, data on patients visiting the outpatient department is not well-characterized. Besides, there is a lack of consensus guidelines on the management of LOA, and there are no established regulations on the use of appetite-stimulating medications. The current study evaluated the effectiveness and safety of an appetite-stimulating medication containing multivitamins (including vitamins B12, B3, and B6), lysine, and zinc in patients with LOA due to acute or chronic illness from South India.

## 2. Methods

### 2.1. Study Design

**Figure 1:**
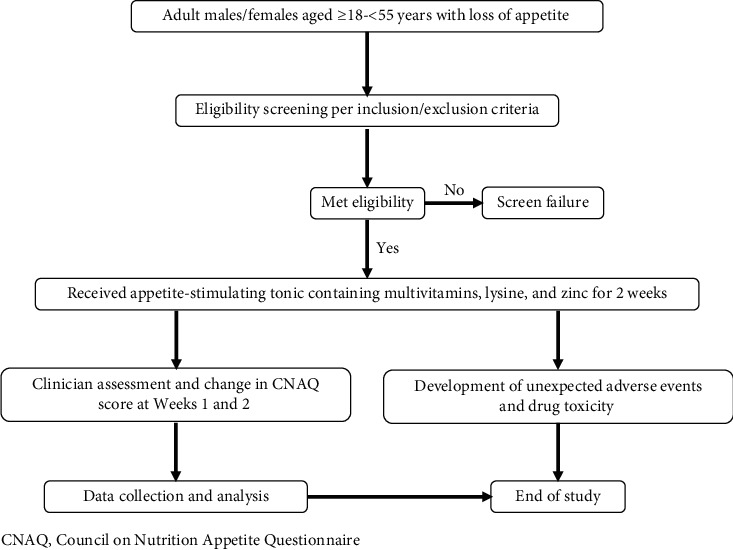
Description of study activities. CNAQ, Council on Nutrition Appetite Questionnaire.

This was an investigator-initiated, single-center, open-label, single-arm study conducted in 50 patients with LOA between August 2019 and October 2019 in Bengaluru, India. Patients were prescribed with an appetite-stimulating medication containing multivitamins, lysine, and zinc (15 mL) twice daily for two weeks before food with a weekly follow-up to assess CNAQ appetite scores (Figure 1).

### 2.2. Study Objectives

The primary objective of the study was to evaluate the effectiveness of the appetite-stimulating medication after completing one week of therapy. The secondary objectives were to evaluate the effectiveness of the medication after two weeks of treatment, to assess the improvement in CNAQ scores after two weeks of treatment, and to evaluate the safety and tolerance of the medication after two weeks of treatment.

### 2.3. Participants

All patients underwent a standardized examination to assess eligibility for the study. The investigator explained the study details to interested patients, and upon patient agreement, voluntary written informed consent was obtained on patient authorization forms. Adult males or females aged 18–55 years, attending the outpatient department with a history of LOA and CNAQ scores <28 due to acute or chronic illness, who were able to understand the study requirements, had undergone physical examination, could abide by restrictions, and return for the required assessments were included in the study.

Pregnant and lactating females, patients taking any antihistaminic or ayurvedic medications for appetite loss during and 7 days before the study, patients with a known history of hypersensitivity to the study drugs used for therapy of appetite loss, and patients with a known condition that may interfere with the absorption or metabolism of study drugs per physician discretion were excluded from the study.

Upon meeting the eligibility criteria, a unique identification number was assigned to each patient. CNAQ scores were assessed at screening visit and at week 1 and week 2 study visits. Treatment compliance was defined as at least 80% adherence to the prescribed dose and frequency, assessed based on patient diaries, and self-reported use at follow-up visits. Subjects with adherence <80% were planned to be excluded from analysis.

### 2.4. Ethics and Compliance

Data completeness and protection of patient safety and rights were ensured. The study was conducted following the principles of the Declaration of Helsinki, International Conference on Harmonization, Good Clinical Practice guidelines, Indian Council of Medical Research, and Drug Controller General of India guidelines. Prior approval of the protocol by the Independent Ethics Committee or Institutional Review Board, submission of any protocol amendments, and all study-related documents were mandatory in compliance with local regulatory requirements.

### 2.5. Study Variables

The primary study variable was the mean change in the CNAQ score from baseline to week 1. The secondary variables included mean change in CNAQ scores from baseline to week 2, frequency and percentages of the CNAQ score at baseline and weeks 1 and 2, and the frequency and percentages of adverse events (AEs), serious adverse events (SAEs), and AEs related to study drug.

### 2.6. Statistical Analysis

Qualitative data were summarized using numbers (*n*) and percentages (%). Continuous variables were presented as mean (standard deviation (SD)) unless otherwise specified. The primary variable was assessed in the intention to treat (ITT) population, which included all patients who were enrolled in the study. The secondary variables were assessed in the safety population, which included all enrolled patients who received at least one dose of the study medication. *P* values were calculated using the paired *t*-test at a 5% level of significance for difference from baseline to week 1 in the CNAQ scores. No formal sample size calculation was performed. Statistical analysis was performed using SAS software.

## 3. Results

### 3.1. Demographics and Patient Baseline Characteristics

A total of 50 patients were screened and enrolled in the study. All patients met the compliance criteria, completed the study, and were included in analysis.

The mean age of patients was 42.12 (9.41) years, with the majority of patients being males (66%). The mean body mass index was 24.67 (3.81) kg/m^2^ (Table 1). In all, 44% of the patients had a medical history of type II diabetes mellitus, and 32% reported with essential hypertension. No relevant family history was reported in the study. A summary of the medical history of patients by acute and chronic illness is given in Table 2. Medications related to comorbid conditions taken by the patients at entry and/or during the study are given in Table 3. All medications used were indicated for preexisting conditions.

### 3.2. Effectiveness Outcomes

**Figure 2:**
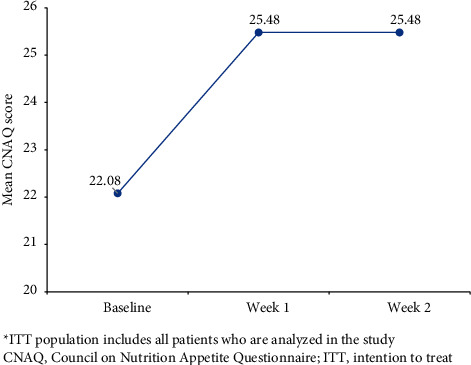
Mean change in CNAQ scores at weeks 1 and 2 from baseline (ITT population)^*∗*^. ^*∗*^ITT population including all patients who are analyzed in the study. CNAQ, Council on Nutrition Appetite Questionnaire; ITT, intention to treat.

A statistically significant (*P* < 0.0001) improvement in the mean CNAQ score was observed at week 1 (25.48 (5.10)) from baseline (22.08 (2.76)), with a mean change of 3.4 (95% CI: 1.92; 4.88; Figure 2). Similarly, a statistically significant (*P* < 0.0001) improvement was observed in the mean CNAQ score at week 2 (25.48 (4.29)) from baseline score of (22.08 (2.76)), with a mean change of 3.4 from baseline (95% CI: 2.13; 4.67; Figure 2).

**Figure 3:**
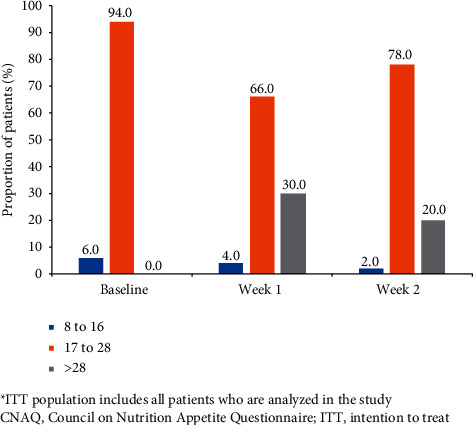
Proportion of patients by CNAQ score ranges (ITT population)^*∗*^. ^*∗*^ITT population including all patients who are analyzed in the study. CNAQ, Council on Nutrition Appetite Questionnaire; ITT, intention to treat.

Majority of the patients had CNAQ scores of 17–28 at baseline (94%), week 1 (66%), and week 2 (78%; Figure 3).

The mean change in the CNAQ scores for patients with acute and chronic illnesses showed statistically significant improvements at week 2 from baseline. For patients with acute illness, a statistically significant improvement was observed in the mean CNAQ score at week 1 (26.75 (3.69), *P* = 0.0256) and week 2 (26.63 (3.46), *P* = 0.0027) from baseline (21.88 (3.31)). The mean change from baseline to week 1 and week 2 was 4.88 (95% CI: 0.79; 8.96) and 4.75 (95% CI: 2.27; 7.23), respectively.

Similarly, for patients with chronic illness, a statistically significant improvement was observed in the mean CNAQ score at week 1 (25.24 (5.33), *P* = 0.0004) and week 2 (25.26 (4.43), *P* < 0.0001) from baseline (22.12 (2.69)). The mean change from baseline at week 1 was 3.12 (95% CI: 1.48; 4.76) and at week 2 was 3.14 (95% CI: 1.69; 4.60).

### 3.3. Safety Outcomes

No events of change in dose or change of the study due to intolerance or adverse event due to the study drug were reported. A total of 3 AEs (vomiting) with mild severity were reported during the follow-up period after the treatment duration was completed. These AEs resolved without any sequelae and were not found to be related to study treatment. No SAEs or deaths were reported in the study.

## 4. Discussion

Appetite-stimulating medications such as dronabinol, megestrol, and mirtazapine are used for weight gain in the outpatient setting; however, there is limited information about overall safety or effectiveness in patients with LOA in the Indian setting. Overall, our study findings in an Indian outpatient setting showed that an appetite-stimulating medication containing multivitamins (including vitamins B12, B3, and B6), lysine, and zinc was a suitable treatment option for the management of patients with LOA. There were no significant safety concerns identified. No serious events involving vomiting and/or uneasiness leading to treatment discontinuation were observed. For acute and chronic patients with LOA, a statistically significant improvement was observed in the mean CNAQ score at weeks 1 and 2. Thus, patients depicted remarkable tolerance to the treatment during the study, thereby suggesting that the appetite-stimulating medication containing multivitamins (including vitamins B12, B3, and B6), lysine, and zinc can be considered as a treatment option for Indian patients with LOA.

The duration of treatment plays a vital role in the efficacy of appetite-stimulating medications. Therapy for at least 2 weeks with appetite-stimulating medications such as megestrol, dronabinol, and mirtazapine has shown benefits in patients with LOA previously [14–17]. This observation is consistent with findings in our study where the treatment duration was for two weeks. Considering that there is no fixed duration established during which an appetite-stimulating medication may show efficacy, it is expected that treatment with such medications for at least two weeks or more may be likely to depict benefits in patients with LOA [17]. In the outpatient setting, the outcomes could be variable considering diverse factors such as patients' due diligence in taking medications and timing of taking medications, which may in turn affect the weight and appetite of the patient. However, in our study, patients with LOA were at least 80% compliant with the prescribed appetite-stimulating medication dose and frequency as assessed from patient diaries and self-reported use at follow-up visits, thereby making our findings relevant to an outpatient setting.

No clear guidelines exist for the management of patients with LOA in India. In the absence of established guidance, assessing symptoms of LOA such as inadequate weight or weight loss in both children and adults could pose a challenge in the outpatient setting. In our study, symptoms in patients were evaluated at the time of study inclusion, i.e., patients with a history of LOA and a CNAQ score of <28 due to acute or chronic illness were included in the study. The majority of the patients with LOA in our study had no relevant family history. However, type II diabetes (44%) and hypertension (32%) were the leading medical histories. These conditions are frequently observed in patients with LOA, especially among the elderly [18]. Thus, there is a need to establish guidelines that take into consideration patients' medical and family histories to achieve better outcomes with the proposed treatments.

Safety, tolerability, and effectiveness of appetite-stimulating medications play a key role in the choice of treatment for patients with LOA. In a recent survey amongst consulting physicians and general practitioners in India, 54% of the physicians preferred multivitamin and multimineral containing appetite-stimulating medications for patients with LOA; 24% and 22% of physicians preferred antihistamine syrup treatment and polyherbal medication, respectively [1]. Compared to the safety observations in our study where vomiting was the only adverse event reported with mild severity and was resolved without any sequelae, other appetite-stimulating medications such as megestrol [14, 19], chronic dronabinol [20], and mirtazapine [21, 22] have shown mild to less severe adverse effects such as dizziness and nausea except for megestrol which may cause severe risks, especially in hospitalized patients [19]. No SAEs were noted in our study, thereby, making the appetite-stimulating medication containing multivitamins, lysine, and zinc a good treatment option for Indian patients with no significant safety concerns.

A CNAQ score of ≤28 indicates a significant risk of at least 5% weight loss within 6 months [8]. In our study, though a statistical significance was observed in CNAQ scores at weeks 1 and 2, the mean CNAQ score was observed to be 25.48, which was lesser than 28, indicating a risk of weight loss in patients with LOA. This could be due to drug-induced suppression of appetite in chronic disease management with certain antidiabetic and antihypertensive medications [23–26]. Despite concomitant medications, the appetite-stimulating medication was well tolerated in our study, thus emphasizing its efficacy. Though beyond the scope of our study, a longer study duration or an increased follow-up would have helped identify the risk intensity in such patients who were administered appetite-stimulating medication containing multivitamins (including vitamins B12, B3, and B6), lysine, and zinc.

The current study evaluated appetite-stimulating medications in an outpatient setting akin to a real-world scenario. Given the open-label nature of the study, there is a potential of bias as there was no blinded outcome assessment. Hence, the knowledge of treatment allocation may introduce a bias that could potentially affect the validity of trial results. There could have been a risk of potential drug interactions with the appetite-stimulating medication. There may have been an increased probability of developing tolerance to appetite-enhancing effects of such medications taken for a prolonged duration. Nevertheless, such trials seem to have higher recruitment rates, and the inclusion of patients is more straightforward [27]. Furthermore, our study did not assess outcomes such as quality of life in patients with LOA to understand the benefit of the appetite-stimulating medication objectively.

## 5. Conclusions

In conclusion, our study provided valuable information regarding the use and effectiveness of an appetite-stimulating medication containing multivitamins (including vitamins B12, B3, and B6), lysine, and zinc among Indian patients with LOA in an outpatient setting. In patients with acute and chronic illnesses, statistically significant improvements were demonstrated on completion of one week and also after two weeks in the mean CNAQ score from baseline. No significant safety concerns were identified. Patients depicted remarkable tolerance to the medication during the study. However, further studies with a larger sample size are needed to validate our study findings.

## Figures and Tables

**Table 1:** Demographics and patient baseline characteristics (safety population)^*∗*^.

Parameter	Overall (*N* = 50)
Gender, *n* (%)	
Male	33 (66)
Female	17 (34)
Age (years)	
Mean (SD)	42.12 (9.41)
Median	43.00
Range	22.00; 53.00
Weight (kg)	
Mean (SD)	66.37 (11.91)
Median	68.00
Range	40.00; 94.00
BMI (kg/m^2^)	
Mean (SD)	24.67 ± 3.81
Median	24.91
Range	14.69; 35.82
Medical history of patients, *n* (%)	
Loss of appetite	50 (100)
Type II diabetes mellitus	22 (44.0)
Essential hypertension	16 (32.0)
Viral fever and hypothyroidism	6 (12.0) each
Vitamin deficiency and allergic bronchitis	5 (10.0) each
Vitamin B12 deficiency	3 (6.0)
Arthritis	2 (4.0)
Acute febrile illness, diabetic foot injury, fever, hyperlipidemia, and vitamin D deficiency	1 (2.0) each

^*∗*^Safety population consisted of all patients who received at least one dose of the study medication. SD, standard deviation.

**Table 2:** Medical history summary according to acute and chronic illness (safety population)^*∗*^.

Medical heading term, *n* (%)	Overall (*N* = 50)
Acute illness	8 (16.0)
Acute febrile illness, loss of appetite, hyperlipidemia	1 (2.0)
Fever, loss of appetite, essential hypertension	1 (2.0)
Viral fever, loss of appetite	2 (4.0)
Viral fever, loss of appetite, allergic bronchitis	1 (2.0)
Viral fever, loss of appetite, arthritis	2 (4.0)
Viral fever, loss of appetite, hypothyroidism	1 (2.0)

Chronic illness	42 (84.0)
Allergic bronchitis, loss of appetite	1 (2.0)
Essential hypertension, loss of appetite	6 (12.0)
Hypothyroidism, loss of appetite	3 (6.0)
Hypothyroidism, loss of appetite, essential hypertension	1 (2.0)
Loss of appetite, essential hypertension	1 (2.0)
Type II diabetes mellitus, loss of appetite	11 (22.0)
Type II diabetes mellitus, loss of appetite, allergic bronchitis	3 (6.0)
Type II diabetes mellitus, loss of appetite, essential hypertension	7 (14.0)
Type II diabetes mellitus, loss of appetite, hypothyroidism	1 (2.0)
Vitamin B12 deficiency, loss of appetite	2 (4.0)
Vitamin D deficiency, loss of appetite	1 (2.0)
Vitamin D deficiency, loss of appetite	4 (8.0)
Vitamin D deficiency, loss of appetite, vitamin B12 deficiency	1 (2.0)

^*∗*^Safety population consisted of all patients who received at least one dose of the study medication.

**Table 3:** Summary of concomitant medication (safety population)^*∗*^.

Class medication name, *n* (%)	Overall (*N* = 50)
Acetic acid derivatives and related substances	5 (10.0)
Aceclofenac	5 (10.0)
Angiotensin II receptor blockers	6 (12.0)
Olmesartan	1 (2.0)
Telmisartan	5 (10.0)
Anilides	5 (10.0)
Paracetamol	5 (10.0)
Selective beta-blocking agents	2 (4.0)
Atenolol	2 (4.0)
Biguanides	22 (44.0)
Metformin	22 (44.0)
Combinations of penicillins, including beta-lactamase inhibitors	1 (2.0)
Amoxicillin/potassium clavulanate	1 (2.0)
Dipeptidyl peptidase 4 inhibitors	8 (16.0)
Teneligliptin	8 (16.0)
Expectorants	3 (6.0)
Guaifenesin	3 (6.0)
HMG-CoA reductase inhibitors	1 (2.0)
Atrovastatin	1 (2.0)
Insulins and analogues for injection (intermediate- or long-acting combined with fast-acting)	1 (2.0)
Insulin	1 (2.0)
Leukotriene receptor antagonists	2 (4.0)
Montelukast	2 (4.0)
Mucolytics	3 (6.0)
Ambroxol	3 (6.0)
Multivitamins with minerals	1 (2.0)
Omega 3 fatty acids, green tea extract, Ginkgo, ginseng, antioxidant, vitamin, minerals	1 (2.0)
Multivitamins (other combinations)	9 (18.0)
Multivitamin	9 (18.0)
Platelet aggregation inhibitors excluding heparin	1 (2.0)
Aspirin	1 (2.0)
Selective beta 2-adrenoreceptor agonists	3 (6.0)
Terbutaline	3 (6.0)
Sulfonylureas	22 (44.0)
Glimepiride	22 (44.0)
Sulfur-containing imidazole derivatives	1 (2.0)
Carbimazole	1 (2.0)
Thiazides	5 (10.0)
Hydrochlorothiazide	5 (10.0)
Third-generation cephalosporins	4 (8.0)
Cefpodoxime	4 (8.0)
Thyroid hormones	5 (10.0)
Thyroxin	5 (10.0)
Vitamin D and analogues	4 (8.0)
Vitamin D3	4 (8.0)

^*∗*^Safety population consisted of all patients who received at least one dose of the study medication ATC, anatomical therapeutic chemical.

## Data Availability

The data used to support the findings of this study are available from the corresponding author upon request.
